# The Diagnostic Value of Pituitary MRI Radiomics Combined with Clinical Features for Central Precocious Puberty in Girls

**DOI:** 10.2174/0115734056498449260721212122

**Published:** 2026-07-25

**Authors:** Yan Li, Wan Chen, Fangying Liu, Qiang Wang, LiJuan Xu, YuLei Zhai, Xuechao Liu, Fan Yang, Shujing Li

**Affiliations:** 1 Hebei Children’s Hospital, Hebei Clinical Medicine Research Center for Children's Health and Diseases, Shijiazhuang, Hebei, China; 2 Hebei Yiling Hospital,, Shijiazhuang, Hebei, 050035, China; 3 Shijiazhuang Maternity and Child Healthcare, Shijiazhuang, Hebei, China

**Keywords:** Central precocious puberty, Idiopathic central precocious puberty, Premature thelarche, Pituitary MRI, Radiomics, Diagnostic model, Clinical features

## Abstract

**Objective:**

To evaluate the diagnostic value of pituitary magnetic resonance imaging (MRI) radiomics features combined with clinical parameters in distinguishing idiopathic central precocious puberty (ICPP) from isolated premature thelarche (PT), and to establish a practical and effective predictive model.

**Materials and Methods:**

A retrospective analysis was conducted on 316 girls presenting with breast development, including 231 with ICPP and 85 with PT, from Hebei Children’s Hospital (internal dataset), and 47 girls (26 with ICPP and 21 with PT) from Shijiazhuang Children’s Hospital (external validation dataset). Clinical models, pituitary MRI radiomics models, and combined clinical-radiomics models were developed. Model performance was evaluated using ROC curves, calibration analysis, and decision curve analysis.

**Results:**

Significant differences were observed between the ICPP and isolated PT groups in body mass index (BMI), bone age index (BAI), follicle-stimulating hormone (FSH), luteinizing hormone (LH), and estradiol (E2) (all *P* < 0.01). The pituitary gland was more frequently protruding in the ICPP group than in the PT group (80.5% vs. 49.5%, *P* < 0.05). In radiomics models, texture features contributed most significantly, with sagittal anterior pituitary features showing the greatest discriminative value. The clinical-radiomics fusion model outperformed individual clinical or radiomics models in predictive performance.

**Discussion:**

Key clinical and radiomics factors for differentiating idiopathic central precocious puberty (ICPP) from premature thelarche (PT) were identified: baseline BMI, BAI, FSH, LH, E2, and PRL are critical clinical indicators (with baseline LH exhibiting superior specificity as an independent diagnostic marker), and multimodal pituitary MRI radiomics analysis (predominantly the RAD-Total model) highlights the anterior pituitary as a dominant predictive feature, with ICPP-related pituitary enlargement attributed to premature hypothalamic-pituitary-gonadal axis (HPGA) activation.

Notably, the clinical-radiomics fusion model (Clinical+RAD-Total) outperforms standalone clinical or radiomics models in diagnostic accuracy, achieving an AUC of 0.944 and considerable clinical net benefit, despite limitations including low sensitivity due to sample imbalance and slightly diminished performance in external validation cohorts.

**Conclusion:**

Clinical-radiomics models demonstrated excellent predictive performance in both internal and external datasets. Among these, the Clinical+RAD-Total model provided the highest diagnostic accuracy and clinical utility.

## 1 INTRODUCTION

Precocious puberty (PP) refers to the condition in which girls develop breast tissue before the age of 7.5 years or experience menarche before the age of 10 years, accompanied by rapid skeletal maturation and accelerated growth [1]. The incidence of PP in pediatric endocrine diseases has shown an increasing trend year by year [2]. Reports from the United States in the 1990s first documented an earlier onset of female puberty [3]. Subsequent studies comparing breast development in girls between 1991–1993 and 2006–2008 revealed a decline in the mean age of breast development from 10.88 years to 9.86 years [4].In Europe, historical analyses demonstrate a substantial decline in menarcheal age, decreasing from approximately 17 years in the early 19th century to about 13 years by the mid-20th century, a reduction of nearly four years [5]. By 1992, the mean menarcheal age had further declined to 12.2 years [1]. Denmark reported a marked increase in PP cases between 1998 and 2017 [6]. A South Korean study [7] also identified a sharp rise in central precocious puberty (CPP) among girls, with incidence rates increasing from 88.9 per 100,000 in 2008 to 1,414.7 per 100,000 in 2020. In China, the median age for reaching the second stage of breast development decreased from 9.78 years in 1993 to 8.26 years in 2010 [8]. PP affects not only physical growth but also psychological well-being and adult height [9]. Early diagnosis and treatment are essential to reduce disease-related harm. PP is classified into three categories based on the activation status of the hypothalamic –pituitary –gonadal axis (HPGA) [10]: Central Precocious Puberty (CPP), peripheral precocious puberty (PPP), and incomplete precocious puberty (IPP). CPP is characterized by premature activation of the HPGA axis, leading to gonadal development and premature epiphyseal closure, and etiologies include central nervous system abnormalities such as tumors or developmental malformations, as well as genetic mutations. Cases without identifiable causes are defined as idiopathic central precocious puberty (ICPP) [11]. The choice of treatment differs according to the subtype of precocious puberty [12]. IPP is further divided into three subtypes: Premature thelarche (PT), precocious pubarche (PP), and precocious menarche (PM), with PT being the most frequent presentation. PT is often associated with obesity or exogenous hormone exposure, and spontaneous resolution may occur after weight reduction or decreased hormone exposure [13]. Although idiopathic central precocious puberty (ICPP) and premature thelarche (PT) present overlapping clinical manifestations, posing diagnostic dilemmas for pediatricians, the gonadotropin- releasing hormone (GnRH) stimulation test remains the gold standard for their differentiation. Nevertheless, this approach is limited by repeated blood sampling, time consumption, high cost, and poor patient and family compliance [14]. Diagnosis of ICPP in females is based on serum estradiol and luteinizing hormone-releasing hormone (LHRH) stimulated luteinizing hormone (LH)/follicle- stimulating hormone (FSH) levels; however, diagnostic cutoffs vary by protocol and stimulus, and no globally accepted thres-holds are available to confirm hypothalamic-pituitary-gonadal axis (HPGA) activation [15]. Serum LH and FSH concen-trations increase progressively with age before menarche [16, 17]. Basal LH, FSH, and the LH/FSH ratio show high discriminative value for distinguishing CPP from non-CPP conditions, with basal LH exhibiting superior specificity [18, 19]. Moreover, urinary LH correlates with serum LH during pubertal development and shows potential for CPP diagnosis, although validated cutoff values are still lacking [20, 21]. To our knowledge, neither the European Society of Paediatric Radiology nor other international bodies have published unified guidelines on imaging in precocious puberty workup, and national guidelines vary [22].

In addition to laboratory testing and ultrasound, the combination of serum hormone measurements and pituitary MRI is essential for the diagnosis of precocious puberty (PP). Traditional imaging has mainly focused on pituitary morphological features. Studies [23, 24] have shown that pituitary size and shape correlate with pubertal development, and pituitary height increases during early infancy and puberty. Pituitary margin bulging has predictive value for central precocious puberty (CPP) but with limited sensitivity. MRI studies have demonstrated that pituitary volumes are significantly larger in girls with idiopathic central precocious puberty (ICPP) than in those with isolated precocious thelarche (PT) [25, 26]. However, the absence of standardized cutoff values requires combination with clinical data to improve diagnostic accuracy.

Radiomics, first introduced by Dutch scholar Lambin in 2012, refers to the high-throughput extraction of quantitative imaging features for advanced analysis [27]. In recent years, radiomics research has expanded rapidly, providing methods to mine reproducible and quantitative information from medical images. Compared with conventional imaging studies, radiomics can reveal tissue heterogeneity and capture microscopic differences that are not visible to the naked eye, thereby offering new insights into clinical diagnosis and prognosis [28, 29]. Recent studies [30] have combined MRI radiomics features with pituitary height measurements to establish predictive models for ICPP, highlighting the value of radiomics in refining pituitary tumor diagnosis and subtype classification. The present study aims to apply radiomics analysis to plain MRI scans of pituitary glands in patients with ICPP and PT. Radiomics-based predictive models will be constructed, along with combined clinical–radiomics models incorporating laboratory data and ultrasound examinations. The performance of each model will be evaluated to identify reliable imaging biomarkers and potential etiological indicators. The goal is to support predictive, prognostic, and diagnostic applications, aligning with precision medicine strategies that have already been widely implemented in oncology research.

## 2 MATERIALS AND METHODS

### 2.1 Study Subjects

A retrospective study was conducted on 231 girls aged 7–10 years (median age: 9.0 years) diagnosed with ICPP at Hebei Provincial Children’s Hospital between February 2023 and March 2025. The diagnosis was made according to the updated 2023 Central Precocious Puberty Diagnosis and Treatment Expert Consensus, which defines PP as breast development before 7.5 years of age or menarche before 10.0 years. In addition, 85 cases of isolated PT were included, with patients aged 6–10 years (median age: 9.0 years, range: 8.0–10.0 years). These cases are consistent with the general clinical understanding that normal breast development in girls typically begins at approximately 9 years of age. For external validation, 47 female patients from Shijiazhuang Children’s Hospital (March 2022 to August 2025) were included, comprising 26 ICPP cases (aged 7–10 years, median age: 9.5 years) and 21 PT cases (aged 6–9 years, median age: 7.0 years). Inclusion criteria: (1) Female patients; (2) Meeting diagnostic criteria for ICPP or PT [1] ; (3) Diagnosis confirmed by GnRH stimulation test; (4) Completed pituitary MRI examination. Exclusion criteria: (1) Current treatment with steroid hormones; (2) Incomplete clinical or imaging data; (3) Central nervous system disorders (*e.g*., brain tumors, encephalitis, trauma, or genetic/metabolic diseases); (4) Presence of glandular tumors (gonadal or adrenal), adrenal hyperplasia, McCune–Albright syndrome, or reproductive tract malformations; (5) Thyroid dysfunction; (6) Poor-quality pituitary MRI images unsuitable for region-of-interest delineation; (7) Interval of more than three days between pituitary MRI and GnRH stimulation test. The study was approved by the Ethics Committee of Hebei Provincial Children’s Hospital, China (Approval No.: 202407-77). All cases from Hebei Provincial Children’s Hospital were randomly divided into a training set (221 cases, including 162 ICPP and 59 PT) and a test set (95 cases, including 69 ICPP and 26 PT) in a 7:3 ratio.

### 2.2 Clinical and Laboratory Examinations

Basic clinical data, including age, height, body weight, body mass index (BMI), and other relevant parameters, were collected from all pediatric patients. Sexual development was evaluated using the Tanner staging system, which categorizes pubertal progression into five stages based on breast development, genital changes, and pubic hair growth in both sexes. Laboratory tests were performed on fasting morning venous blood samples, and results were recorded for FSH, LH, PRL, estradiol (E2), tumor markers, and thyroid function. These indicators are essential for assessing pubertal onset, evaluating the function of the HPGA, and excluding potential endocrine disorders. Skeletal development was assessed by anteroposterior X-ray digital radiography (DR) of the left wrist. Bone age was evaluated using the GP method. In addition, an AI-assisted evaluation method incorporating the Tanner–Whitehouse (TW) scoring system was applied. The TW3 system, an updated version of TW2, retains the original scoring method while adjusting maturity scores and reference values based on more recent population samples. These parameters provide quantitative indicators of skeletal maturation relative to chronological age and are widely applied in evaluating pubertal development.

### 2.3 GnRH Stimulation Test

Serum LH and FSH concentrations were measured using an immunochemiluminescence assay (Cobas e 602, Roche, Switzerland). Fasting venous blood samples were collected between 8:00 AM and 9:00 AM, with assay sensitivities set at 0.01 mIU/mL for LH and 0.05 mIU/mL for FSH. Trilupirulin was administered subcutaneously at a dose of 2.5 μg/kg (maximum dose 100 μg). Blood samples were obtained at 0, 30, 60, and 90 minutes after administration to measure LH and FSH concentrations. The peak ratio of LH to FSH (LH/FSH) was subsequently calculated. Subjects with a peak LH level ≥5.0 U/L and an LH/FSH ratio ≥0.6 were classified as ICPP, whereas those not meeting these criteria were categorized as PT [1].

### 2.4 Pituitary MRI Examination

For internal data (including training and test sets), pituitary MRI images were aFor internal datasets (training and test cohorts), pituitary MRI examinations were performed using Philips Achieva 1.5T, Ingenia Ambition S 1.5T, or Siemens Skyra 3.0T scanners, equipped with either an 8-channel head coil or a 20-channel head–neck coil. For the external validation cohort, imaging was performed with a Philips Prodiva 1.5T scanner using an 8-channel head coil. All pediatric subjects were placed in the supine position with the head oriented forward, the body aligned with the examination table, shoulders positioned against the coil, and hands resting laterally. The head was stabilized at the mid-forehead line using a sponge pad. Detailed scanning parameters are summarized in Table **1**.

### 2.5 Radiomics

#### 2.5.1 Hypophyseal Region of Interest Segmentation

Pituitary regions of interest (ROIs) were delineated using the Shenrui Medical Multimodal Research Platform, version 2.2.1 (Beijing Shenrui Bolian Technology Co., Ltd., Beijing, China; https://keyan.deepwise.com). Two radiologists (Reader A with 8 years and Reader B with 12 years of diagnostic experience) independently performed ROI segmentation. When delineation discrepancies exceeded 5%, a senior radiologist with 30 years of neuroimaging diagnostic experience (Reader C) adjudicated the final boundaries to ensure delineation accuracy and data validity. To evaluate the reproducibility and robustness of radiomics feature extraction, 60 cases were randomly selected from the internal dataset. These cases were re-segmented by the two radiologists, and the intraclass correlation coefficient (ICC) was calculated.

The anterior and posterior pituitary lobes were segmented using sagittal and coronal T1-weighted images (T1WI), respectively, based on their characteristic signal intensities. In normal adolescents, the anterior pituitary typically demonstrates isointensity relative to the brainstem, whereas the posterior lobe exhibits high signal intensity [31] (Figs. **1** and **2**).

#### 2.5.2 Radiomics Preprocessing

To minimize variability in image pixel intensities introduced by differences in scanners and magnetic field strengths, pituitary MRI images underwent preprocessing before feature extraction. Two key steps were implemented: resampling and normalization. During the resampling process, the B-spline interpolation algorithm was applied to standardize the spatial resolution of all images to an isotropic voxel size of [1, 1, 1]. This approach preserves spatial consistency across datasets by generating smooth interpolated values, thereby ensuring comparability of spatial information during subsequent analysis. For intensity normalization, a linear scaling method was employed to adjust pixel values to a uniform range of (0, 100), effectively reducing grayscale variation and maintaining consistency in image intensity distributions. These preprocessing steps provided a standardized foundation for accurate and reproducible radiomics feature extraction in subsequent analyses.

#### 2.5.3 Feature Extraction and Feature Screening

Radiomics feature extraction was performed separately for the anterior and posterior pituitary lobes in all samples, yielding 1,687 features per ROI. The extracted features included first-order statistics, morphological descriptors, and texture parameters. All features were standardized using Z-score normalization to achieve a mean of zero and a variance of one. To ensure robustness, features with ICC below 0.9 were excluded. Redundancy was further reduced by removing features with Spearman correlation coefficients greater than 0.9 The remaining features were subjected to dimensionality reduction using the least absolute shrinkage and selection operator (LASSO) regression, thereby retaining the most informative variables for subsequent model construction.

#### 2.5.4 Model Construction

Based on the clinical and radiomics features identified in Section 2.5.3, multiple logistic regression models were constructed. Three single-sequence radiomics models were developed: the RAD-AL1 model, incorporating radiomics features of the anterior pituitary on sagittal T1WI; the RAD-AL2 model, including features from the anterior pituitary on coronal T1WI; and the RAD-PL model, based on features from the posterior pituitary on sagittal T1WI. For feature integration, radiomics features from anterior and coronal T1WI were combined after ICC filtering, followed by Spearman correlation analysis and LASSO regression to construct the RAD-AL model. Similarly, features from the anterior and posterior pituitary T1WI were merged after ICC filtering, correlation analysis, and LASSO selection to establish the RAD-Total model. Radiomics scores derived from selected features were converted into a composite Radscore using a radiomics scoring formula. Clinical features, including BMI, BAI, FSH, LH, and E2, were integrated with the Radscore. After Spearman correlation analysis and LASSO selection, these parameters were incorporated into the Clinical+RAD-Total model. The overall workflow of model development is illustrated in Fig. (**3**).

#### 2.5.5 Statistical Analysis

All statistical analyses were conducted using the Shenrui Medical Multimodal Research Platform version 2.2.1 (Beijing Shenrui Bolian Technology Co., Ltd., China). The ICC was calculated to assess intra-observer and inter-observer consistency of radiomics feature extraction. For clinical factor analysis, continuous variables with a normal distribution were compared between groups using independent-sample t-tests, whereas non-normally distributed variables were analyzed using the Wilcoxon rank-sum test. Categorical variables were assessed using either the chi-square test or Fisher’s exact test, as appropriate. To evaluate model performance, receiver operating characteristic (ROC) curves were plotted, and the area under the curve (AUC), accuracy (ACC), sensitivity (SEN), and specificity (SPE) were calculated. Comparisons of model performance were conducted using DeLong’s test. In addition, decision curve analysis (DCA) was performed to estimate clinical net benefit (NB) across different threshold probabilities.

## 3 RESULTS

### 3.1 Analysis of Clinical Characteristics and Establishment of Clinical Models

The clinical data analysis between the ICPP and PT groups (including age, BMI, BAI, FSH, LH, PRL, and E2) is presented in Table **2**. Significant differences were observed in BMI, BAI, FSH, LH, and E2 levels between the two groups (*P* < 0.01). In addition, age and progesterone levels also showed statistically significant differences (*P* < 0.05). However, PRL levels did not differ significantly between groups (*P* > 0.05). Overall, patients in the ICPP group demonstrated markedly elevated baseline hormone levels compared to those in the PT group. Furthermore, increased BMI and advanced bone age were more prominent in the ICPP group, suggesting that accelerated skeletal maturation may lead to premature termination of growth and potentially compromise final adult height.

Clinical indicators that showed significant differences between the ICPP and PT groups (*P* < 0.01), including BMI, BAI, FSH, LH, and E2, were incorporated into the logistic regression model. The corresponding weight coefficients for each variable are summarized in Table **3**.

The AUC, ACC, SEN and SPE of the clinical model in the training set and test set are shown in Table **4**.

### 3.2 Establishment of Pituitary MRI Morphology and Radiomics Model

#### 3.2.1 Pituitary Morphology

The pituitary gland was classified into three types: concave, flat, and bulging [31], as shown in Table **5**. In both the ICPP and PT groups, bulging morphology was the most common. Notably, the prevalence of bulging pituitaries in the ICPP group (80.5%) was significantly higher than in the PT group (49.5%), and the difference reached statistical significance (*P* < 0.05).

#### 3.2.2 Establishment of a Radiomics Model

From the ROI delineated on the T1=WI sagittal view of the anterior pituitary, the T1WI coronal view of the anterior pituitary, and the T1WI sagittal view of the posterior pituitary, a total of 1,687 radiomics features were extracted, including first-order, shape, and texture features. The consistency of extracted features was evaluated by ICC, based on ROI delineation performed independently by two radiologists. Features with ICC values greater than 0.9 were considered to have good reproducibility. After excluding features with ICC values below 0.9, 654, 950, and 501 features remained for the anterior pituitary sagittal view, anterior pituitary coronal view, and posterior pituitary sagittal view, respectively.

Subsequent feature selection was performed using Spearman correlation analysis and LASSO regression, with the optimal lambda value determined through cross-validation. The retained features were incorporated into logistic regression classifiers to establish radiomics and combined clinical–radiomics models. The RAD-AL1 model (anterior pituitary sagittal plane) retained 7 features (2 first-order, 1 shape, and 4 texture features), the RAD-AL2 model (anterior pituitary coronal plane) retained 10 features (1 first-order, 2 shape, and 7 texture features), and the RAD-PL model (posterior pituitary sagittal plane) retained 7 features (3 first-order and 4 texture features). The RAD-AL model, integrating sagittal and coronal anterior pituitary features, retained 8 features (1 first-order and 7 texture features), consisting of 4 sagittal and 4 coronal features. The RAD-Total model, combining anterior and posterior pituitary features, retained 13 features (3 first-order, 2 shape, and 8 texture features), including 7 sagittal anterior pituitary, 4 coronal anterior pituitary, and 2 sagittal posterior pituitary features Table **6**. These results are illustrated in Figs. (**4** and **5**). All radiomics model scores were calculated using the Rad-score formula:


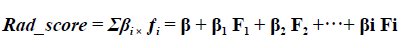


Here, fi represents the projection omics feature i, and ßi represents the weight coefficient corresponding to the feature.

All models retained the largest proportion of texture features, highlighting the critical role of texture parameters in predictive performance. Among them, the RAD-Total model preserved the greatest number of features derived from the T1WI sagittal view of the anterior pituitary gland, suggesting that this anatomical region contributes substantially to improving prediction accuracy.

#### 3.2.3 Performance Evaluation and Comparison of Imagomics Models

The predictive performance of the RAD-AL1, RAD-AL2, RAD-AL, RAD-PL, and RAD-Total models was evaluated using AUC, ACC, SEN, and SPE, as summarized in Table **7**. In the training set, the RAD-Total model achieved the highest predictive performance, with an AUC of 0.846 (95% CI: 0.7848–0.9078), surpassing all other models constructed solely from radiomics features. Its corresponding ACC, SEN, and SPE values were 0.81, 0.3729, and 0.9691, respectively. In the training set, the RAD-Total model achieved the highest predictive performance, with an AUC of 0.846 (95% CI: 0.7848–0.9078), surpassing all other models constructed solely from radiomics features. Its corresponding ACC, SEN, and SPE values were 0.81, 0.3729, and 0.9691, respectively. The Delong test analysis further compared the RAD-Total model with the RAD-AL1, RAD-AL2, RAD-AL, and RAD-PL models (Table **8**). Significant differences were observed in the training set between the RAD-Total model and all other radiomics models. In the validation set, the RAD-Total model showed a significant difference compared with the RAD-PL model, whereas no significant differences were found when compared with the RAD-AL1, RAD-AL2, or RAD-AL models. Overall, these results demonstrated that the RAD-AL1, RAD-AL, and RAD-Total models provided the strongest predictive performance in differentiating ICPP from PT. By contrast, the RAD-PL model consistently showed the weakest predictive capability. These findings suggest that radiomics features derived from the T1WI sagittal view of the anterior pituitary contribute more substantially to model performance than those from the coronal anterior pituitary view, whereas features from the posterior pituitary sagittal view offer minimal additional value.

#### 3.2.4 Establishment and Comparison of Clinical Radiomics Fusion Models

Using logistic regression classifiers, five fusion models were constructed by integrating all clinical variables with the Rad-score derived in Section 3.2.2: Clinical + RAD-AL1, Clinical + RAD-AL2, Clinical + RAD-AL, Clinical + RAD-PL, and Clinical + RAD-Total. Model performance was assessed in the training, test, and external validation sets, with ROC curve analysis yielding AUC, ACC, SEN, and SPE values (Table **9**). Compared with the radiomics-only models described in Section 3.2.3, the incorporation of clinical features consistently improved predictive performance. In the test set, the AUC values increased from 0.844, 0.799, 0.867, 0.598, and 0.854 to 0.9052, 0.9114, 0.914, 0.909, and 0.944, respectively. Results from the external validation set demonstrated similar improvements. Notably, the Clinical + RAD-Total model achieved the highest AUC among all models, indicating superior discriminative ability. Moreover, all fusion models outperformed the clinical model alone, which achieved an AUC of 0.844 in the test set. These findings suggest that integrating radiomics features with clinical parameters enhances diagnostic accuracy and provides more reliable predictive models for differentiating ICPP from PT. •

Studies have shown that integrating clinical and radiomics data into a unified model improves classification accuracy beyond the performance of individual clinical or radiomics models. The ROC curves, decision curves, and calibration curves for the five clinical–radiomics models in the training and test datasets are presented in Fig. (**6**), as reported in previous studies discussing the use of ROC analysis in clinical prediction models. Among these models, the Clinical + RAD-Total model demonstrated the strongest predictive capability for distinguishing intracranial pressure (ICP) from proptosis, achieving higher accuracy than other models. However, its SEN remained relatively low.

### 3.3 Correlation of Features, Swarm Plots, Column Charts, ROC Curves, Calibration, and Clinical Decision Utility Assessment in the Clinical+RAD-Total Model

The Clinical+RAD-Total model incorporated six features: Rad-score, BMI, BAI, FSH, LH, and E2. Correlation analysis among these features is presented in Fig. (**7**), while the impact of each feature on model output is shown in Fig. (**8**). The results demonstrate that the Rad-score exhibits the largest variation in SHAP values, indicating its dominant influence on prediction accuracy. High feature values, represented by red dots, correspond to positive SHAP values, highlighting their positive contribution to model predictions.

The retained features and weight coefficients after feature screening are shown in Table **10**. BMI, BAI, and FSH were identified as contributing factors in the model.

The clinical-RAD-Total model, which integrates clinical quantitative factors (BMI, BAI, FSH) with radiomics characteristics, is illustrated in Fig. (**9**). Presented as a nomogram, this visualization incorporates BMI, BAI, FSH, and the Rad-score derived from the RAD-Total model into a binary logistic regression framework. The total score calculated from individual patient characteristics corresponds directly to the probability of ICPP occurrence. The probability of clinical outcomes predicted by multiple variables is represented by scored line segments.

The ROC curves of the clinical-RAD-Total model in the training set and the verification set are shown in Figs. (**10** and **11**).

The DCA of the Clinical+RAD-Total model, as shown in Fig. (**12**), illustrates clinical net benefit performance across different thresholds. The analysis indicates that the model provides greater net benefit for clinical decision-making compared with scenarios in which all patients are classified as ICPP or PT, as represented by “All” and “None” in the figure. In the training set, the clinical net benefit remains positive when the predicted probability is below approximately 86%. In contrast, in the test set, the benefit persists when the probability is below about 90%, underscoring the strong clinical applicability. Calibration curves for the training and test sets are displayed in Figs. (**13** and **14**), respectively, to assess the agreement between predicted and actual probabilities.

## 4 DISCUSSION

The present study retrospectively analyzed female patients diagnosed with ICPP and premature thelarche (PT) using GnRH stimulation tests as part of routine clinical assessment. By integrating clinical quantitative parameters with radiomics analysis of pituitary MRI, specific biomarkers were identified that enabled differentiation between these two conditions. A predictive model was subsequently established, providing potential clinical value for prognostic evaluation. Logistic regression incorporating BMI, BAI, baseline hormone levels, and pituitary MRI radiomics features yielded a model with strong diagnostic performance, which was consistently validated across the training set, test set, and external cohort.

### 4.1 Analysis of Clinical Factors Related to PP

The clinical model retained BMI, BAI, FSH, LH, and E2 as key features. The mean or median values of these parameters were consistently higher in the ICPP group than in the PT group. With respect to BMI, a strong biological association exists between body weight and the onset of puberty. In idiopathic central precocious puberty (ICPP), premature activation of the hypothalamic-pituitary-gonadal axis (HPGA) and increased sex steroids accelerate skeletal growth, advancing bone age and promoting early epiphyseal closure. While this results in a transient height gain, it often compromises final adult height [32]. In contrast, peripheral precocious puberty (PT), without HPGA activation, shows no significant bone age advancement, aligning with the current observations. RFSH levels were also elevated in the ICPP group. A comparison between 30 ICPP patients and 30 normally developing children showed significantly higher baseline serum FSH levels in the ICPP cohort, consistent with the present results. Multiple studies confirmed that baseline LH has higher specificity than FSH when used as an independent diagnostic marker, a finding that also supports the current analysis [15, 33, 34]. Regarding PRL, both ICPP and PT patients presented clinical signs of breast development and elevated PRL, and the mean PRL level was higher in the ICPP group than in the PT group, which was different from Lee *et al*. said non-CPP girls had a higher prevalence of elevated PRL levels (>17.9 ng/mL) [35].

### 4.2 Analysis of Radiomics and Pituitary Morphology Factors Related to PP

Multimodal MR images (T1WI coronal and sagittal views) were used to construct stable and interpretable radiomics models. Among the logistic regression classifiers developed for RAD-AL1, RAD-AL2, RAD-AL, RAD-PL, and RAD-Total, the AUC values ranked as RAD-Total > RAD-AL > RAD-AL1 > RAD-AL2 > RAD-PL. The RAD-Total model preserved seven features from the T1WI sagittal view of the anterior pituitary, four from the T1WI coronal view of the anterior pituitary, and two from the T1WI sagittal view of the posterior pituitary, highlighting the anterior pituitary as the dominant contributor to PP classification. The importance of the anterior pituitary may be attributed to fundamental differences in embryologic origin, anatomy, and vascular supply between the anterior and posterior lobes. The anterior pituitary arises from cystic protrusions of oral epithelial tissue, whereas the posterior pituitary originates from neural tissue..Functionally, the anterior pituitary is a major hormone- producing gland that secretes LH, FSH, and other hormones, whereas the posterior pituitary, as an extension of the hypothalamus, stores but does not synthesize hormones. These structural and functional distinctions underscore the critical role of the anterior pituitary in the development of PP. Imaging biomarkers retained in the models further reinforce the significance of pituitary morphology. For example, the original shape minor axis length on the T1WI coronal view and the major axis length on the T1WI sagittal view of the anterior pituitary reflect the short- and long-axis dimensions of the gland, indicating a strong association between pituitary morphology and ICPP.

Analysis of pituitary MRI images showed that 80.5% of patients in the ICPP group presented with pituitary enlargement. Previous studies consistently demonstrated that pituitary height increases progressively with pubertal development [24-27]. In ICPP, premature activation of the hypothalamic-pituitary-gonadal axis (HPGA) disrupts the balance between sex hormone stimulators and inhibitors, thereby promoting pituitary proliferation. This mechanism accounts for the enlargement of the anterior pituitary observed in the present study, in agreement with published findings.

### 4.3 Clinical Radiomics Fusion Model Evaluation

The clinical-radiomics fusion model demonstrated superior predictive performance for distinguishing ICPP from PT compared with standalone clinical or radiomics models. Among the fusion models, the Clinical+RAD-Total model achieved the best predictive efficacy. When comparing the Clinical, RAD-Total, and Clinical+RAD-Total models, the Clinical model yielded an AUC of 0.844 (95% CI: 0.7487–0.9391) but a SEN of only 0.1583, underscoring its limited ability to identify ICPP and PT patients using clinical variables alone. The RAD-Total model showed improved diagnostic capability with an AUC of 0.854 (95% CI: 0.7786–0.9293) and SEN of 0.2692, indicating a stronger capacity to detect true positives. The Clinical+RAD-Total model further outperformed both, achieving an AUC of 0.944 (95% CI: 0.902–0.9854), an accuracy of 0.8632, and a SEN of 0.3846. Specificity remained comparable across models, confirming that the fusion model enhanced prediction without compromising false-positive rates. DCA showed that the Clinical+RAD-Total model offered a clear net benefit for clinical decision-making compared with strategies that classified all patients as either ICPP or PT (as illustrated in the figure with the “All” and “None” categories). In the training set, the model yielded a positive net benefit when the predicted probability was below approximately 86%. In the test set, the benefit persisted when the probability was below about 90%. These results highlight the strong clinical applicability of the model. Integration of clinical quantitative factors related to PP with multimodal pituitary MRI radiomics features provided more comprehensive diagnostic information, thereby enhancing predictive performance. Zou *et al*. [30] constructed four machine learning models for ICPP prediction, including radiomics-only, comprehensive imaging (pituitary MRI radiomics, uterine/ovarian ultrasound, and X-ray bone age), clinical-only, and multimodal models. Their best-performing multimodal model achieved an AUC of 0.866, while the approach adopted in the present study, which combined radiomics, clinical data, and ultrasound features, achieved higher predictive accuracy and offered a more comprehensive framework for patient evaluation.

However, the SEN values of the clinical models, imaging omics models, and fusion models remained relatively low. One possible explanation is the lower incidence of PT in the cohort, which led to an imbalanced sample distribution. In addition, the AUC values in the external validation sets were slightly lower than those observed in the test set, likely due to smaller sample sizes and uneven data distribution in the validation cohorts.

## 5 INNOVATIONS

(1) Pituitary MRI plain scan images were used to establish independent radiomics models for the anterior and posterior lobes. A combined radiomics model was subsequently developed by integrating features from both lobes. The study not only evaluated the predictive performance of anterior and posterior pituitary radiomics in differentiating ICPP from PT but also incorporated comprehensive radiomics features from the entire pituitary gland. This dual strategy enabled a more thorough analysis and significantly improved the overall model performance.

(2) A clinical–radiomics fusion model was constructed by integrating clinical risk factors with radiomics features. This fusion model demonstrated superior predictive accuracy and generalizability, providing substantial value for the identification of ICPP in clinical practice.

(3) A multicenter design was adopted to further verify the stability and generalizability of the proposed models, strengthening the robustness of the study findings.

## 6 LIMITATIONS

(1) The retrospective design of the study may introduce selection bias.

(2) The relatively small sample size limited the robustness of the findings. Larger cohorts are needed to construct more reliable predictive models and to verify their performance.

(3) The SEN of the Clinical+RAD-Total model was relatively low, which may be explained by the small sample size in the PT group, the larger sample size in the ICPP group, and the imbalance between positive and negative cases. Moreover, the AUC values of all models in the external validation set were slightly lower than those in the test set, possibly due to the limited number of cases, uneven data distribution, and differences in feature representation.

## 7 FUTURE RESEARCH DIRECTION

(1) Current delineation of the pituitary ROI mainly relies on semi-automatic tracing combined with manual adjustments. Given the small size and complex structure of the pituitary gland, these methods may increase error rates and depend heavily on subjective judgment. Automated segmentation techniques remain insufficient. Future studies should focus on developing more accurate automated segmentation methods, particularly using deep learning and interactive approaches, to improve both research and clinical applications.

(2) The small size of the pituitary gland poses challenges for imaging. Conventional MRI protocols with a slice thickness of 2–3 mm may fail to capture detailed structural information. Further studies on thin-slice pituitary MRI scanning are warranted to enhance diagnostic accuracy.

(3) Increasing the sample size and exploring diverse algorithmic modeling strategies may significantly improve predictive performance and strengthen model generalizability.

## CONCLUSION

Among radiomics-only models, the RAD-Total model achieved superior predictive performance, showing lower RMSE, higher F1 scores, and robust generalizability. Radiomics information derived from the anterior pituitary contributed substantially to model prediction. Integration of clinical and radiomics features through fusion modeling further improved diagnostic and prognostic performance beyond that of either modality alone. Within these models, the Clinical+RAD-Total model demonstrated outstanding predictive efficacy, with consistent performance across internal and external datasets, supporting its reproducibility and potential for clinical translation.

## Figures and Tables

**Fig. (1):**
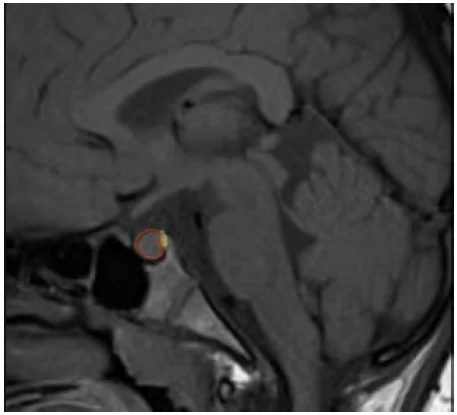
Sagittal region of interest delineated in T1WI (red for anterior pituitary and yellow for posterior pituitary).

**Fig. (2):**
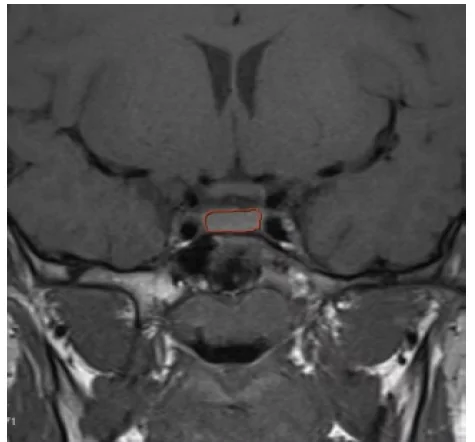
T1WI anterior pituitary area of interest in the coronal view (red marked as anterior pituitary).

**Fig. (3):**
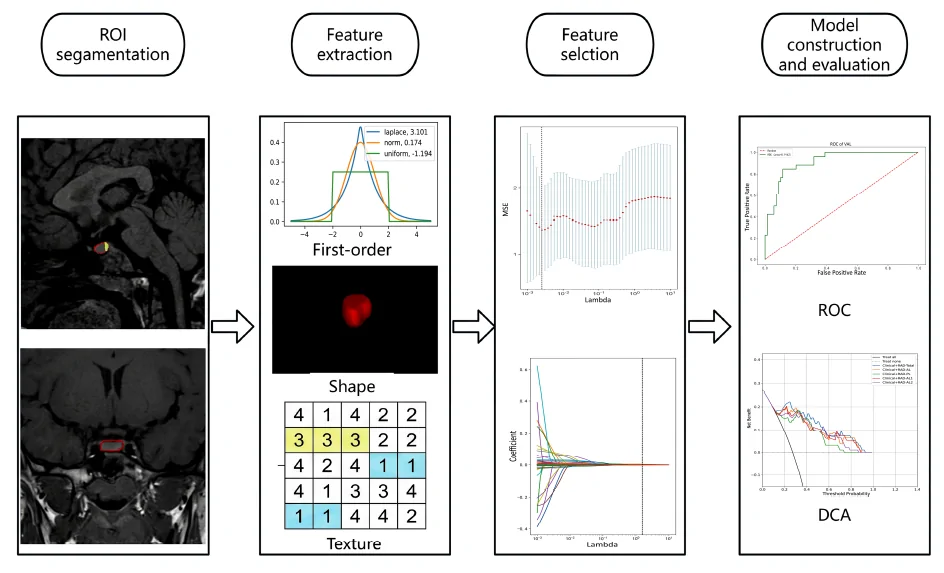
Flow chart of image omics model construction.

**Fig. (4):**
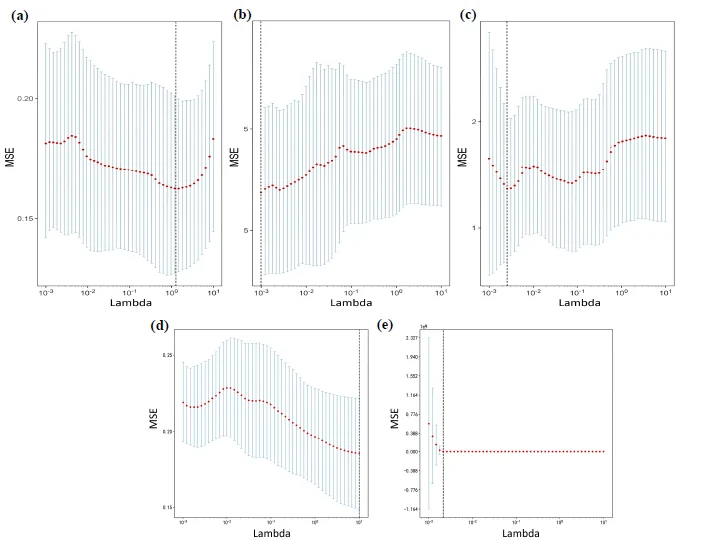
Radiomics feature selection using the LASSO regression algorithm. Panels (**a**-**e**) show the relationship between residual deviance and the log(λ) value, as well as the mean squared error, for the RAD-AL1, RAD-AL2, RAD-AL, RAD-PL, and RAD-Total models, respectively.

**Fig. (5):**
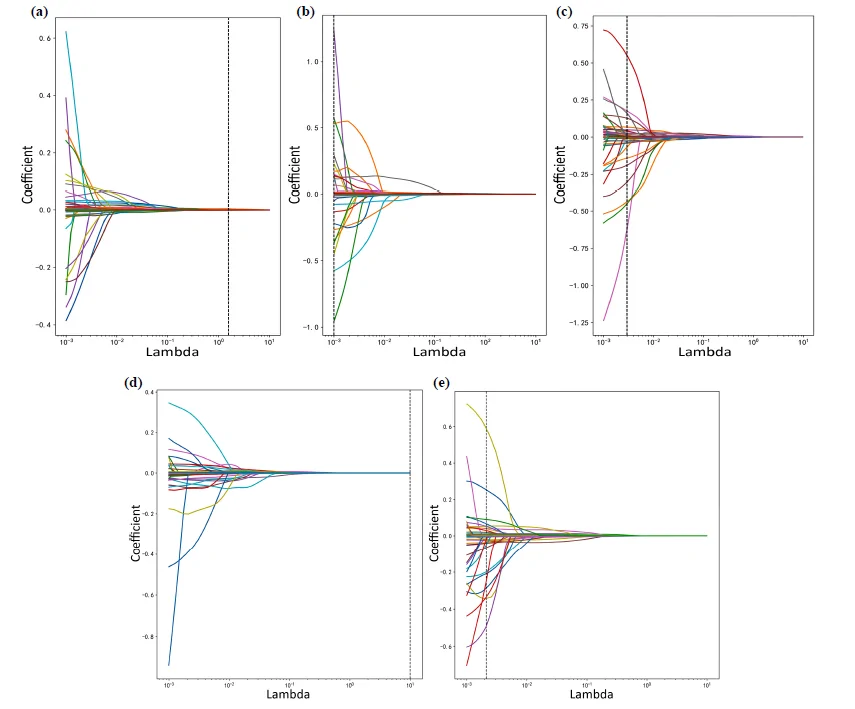
Coefficient profiles of radiomics features for different λ values in the LASSO regression analysis (**a**-**e**). From top to bottom, the plots correspond to the RAD-AL1, RAD-AL2, RAD-AL, RAD-PL, and RAD-Total models.

**Fig. (6):**
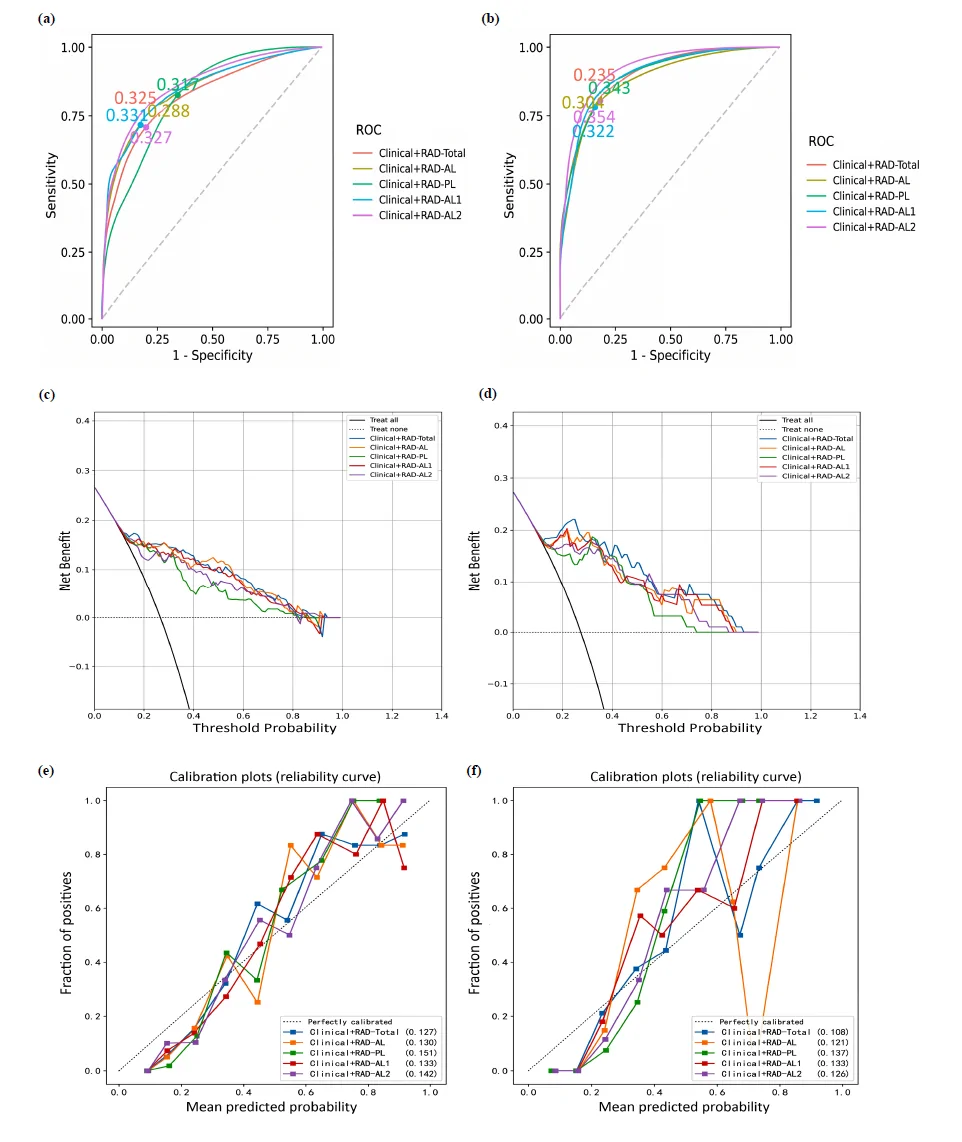
ROC, decision, and calibration curves of the clinical–radiomics models. Panels **a**, **c**, and **e** represent the ROC curve, decision curve, and calibration curve of the training set, respectively; panels **b**, **d**, and **f** represent the corresponding curves of the test set.

**Fig. (7):**
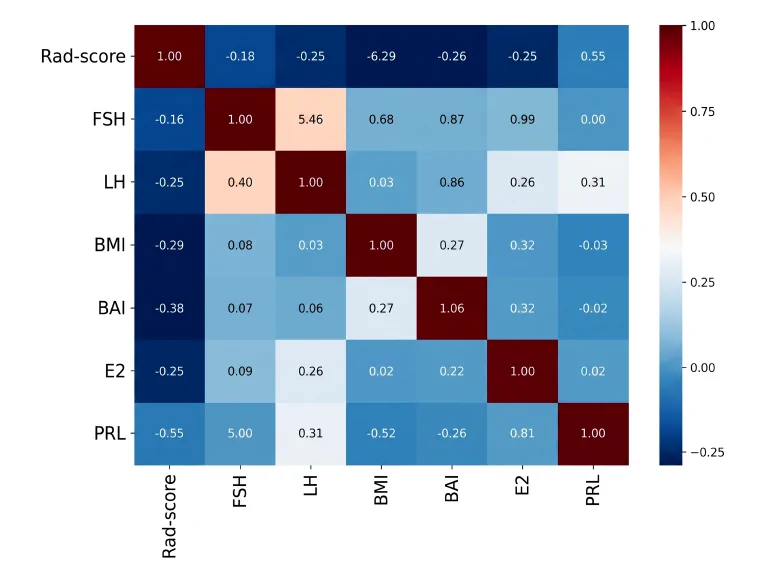
Feature correlation matrix (each row and column represent a feature, and the color of each cell indicates the degree of correlation between two features).

**Fig. (8):**
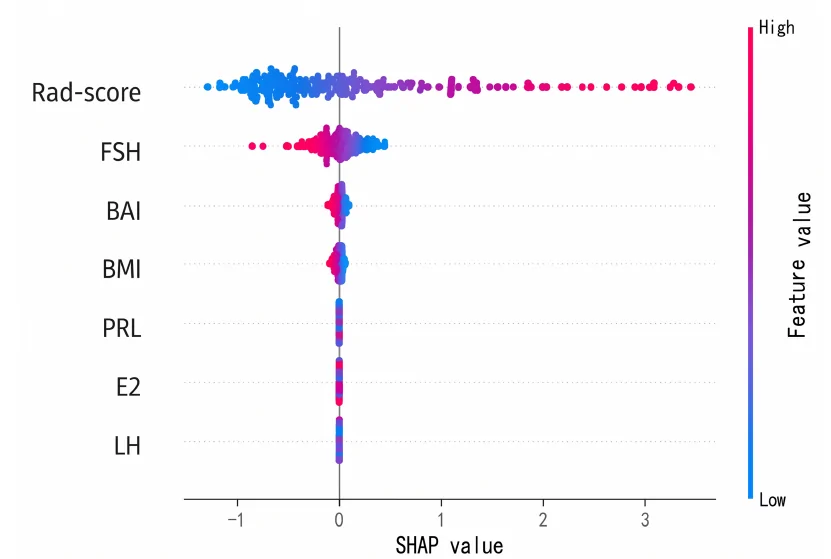
SHAP value swarm plot of features (the horizontal axis represents the magnitude of the SHAP value, reflecting the influence of each feature on the model output, while the vertical axis corresponds to different features).

**Fig. (9):**
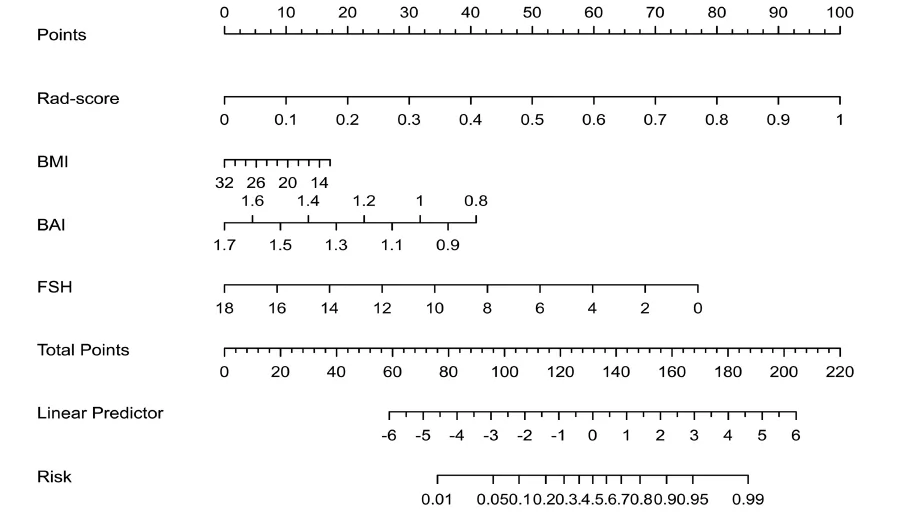
Column plot of clinical-RAD total model.

**Fig. (10):**
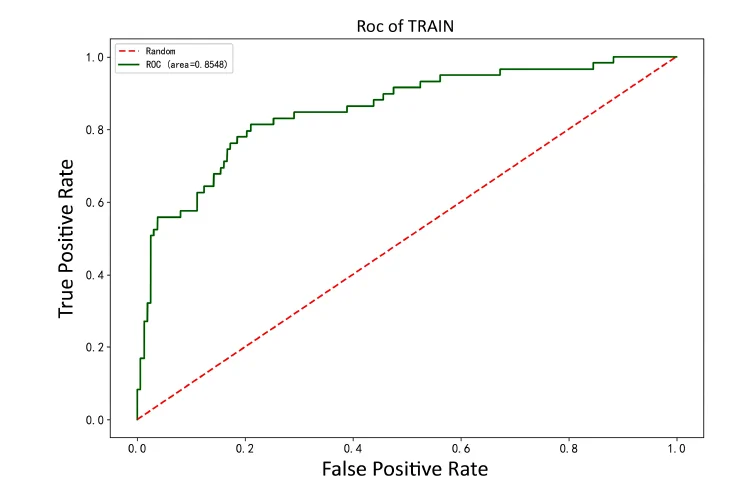
ROC curve of the clinical-RAD-Total model training set.

**Fig. (11):**
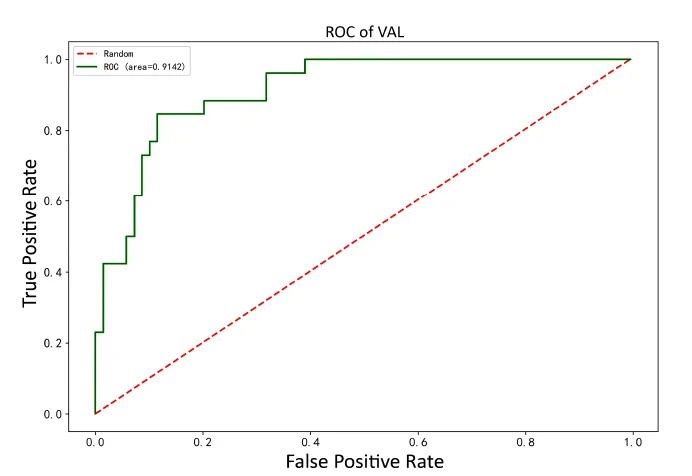
ROC curve of the clinical-RAD-Total model validation set.

**Fig. (12):**
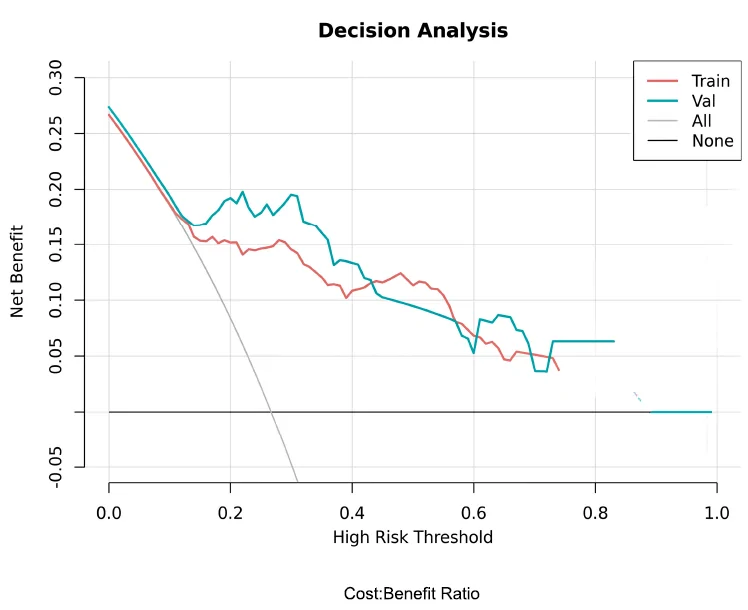
Decision analysis curve of clinical+RAD-Total model.

**Fig. (13):**
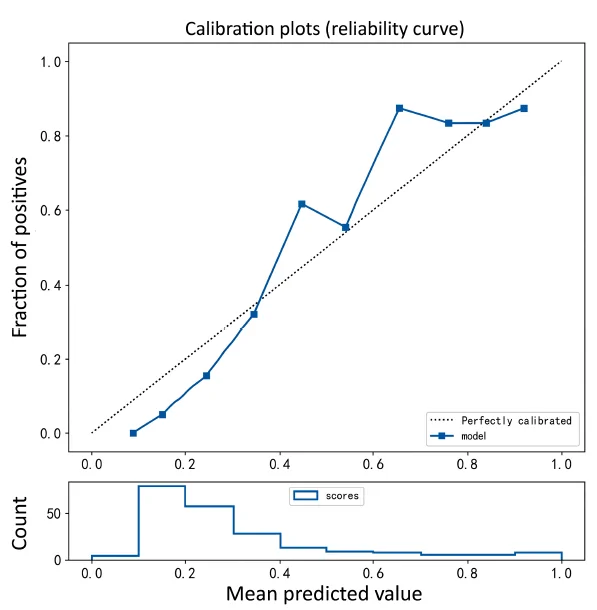
Calibration curve of clinical+RAD-Total model training set.

**Fig. (14):**
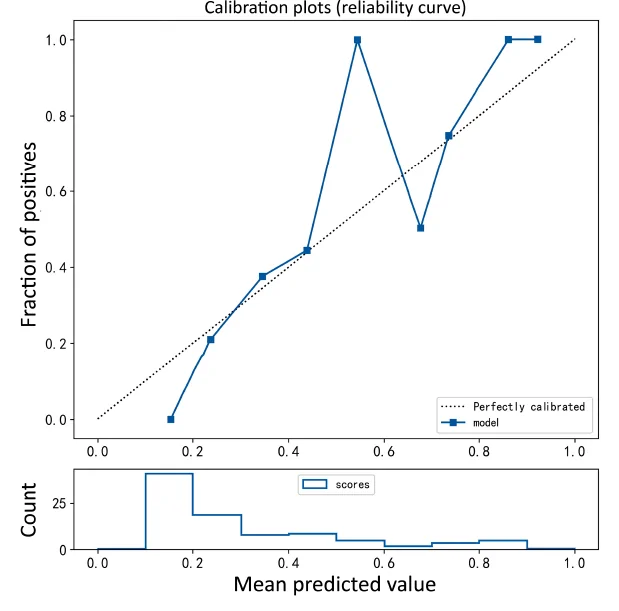
Calibration curve of clinical + RAD-Total model validation set.

**Table 1:** Pituitary MRI equipment and scanning parameters.

**Data Set**	**Unit Type**	**Order**	**TR/TE**	**FOV(mm^2^)**	**Reconstructing the Matrix**	**Depth (mm)**
Internal Data Set	Philip Achieva 1.5T	T_1_Sag	550/15	140×140	256×256	2.5
		T_1_Cor	550/15	140×140	256×256	2.5
	Philip Ingenia Ambition S 1.5T	T_1_Sag	550/15	140×140	256×256	2.5
		T_1_Cor	550/15	140×140	256×256	2.5
	Siemens Skyra 3.0T	T_1_Sag	550/9.5	140×140	256×256	2
		T_1_Cor	550/9.5	140×140	256×256	2
External Validation Set	Philip Prodiva 1.5T	T_1_Sag	500/15	150×150	200×197	3
		T_1_Cor	500/15	140×180	200×225	3

**Table 2:** Clinical data analysis between the IPPC group and the PT group.

-	**ICPP (N=231)**	**PT (N=85)**	**P price**
Age	9.000(9.000-10.000)	9.000(8.000-10.000)	0.048*
BMI index number	20.285±3.195	18.429±2.987	0.000**
Age at bone age	1.271±0.108	1.201±0.147	0.000**
Ovulation-stimulating hormone FSH (IU/L)	5.739±2.067	4.457±1.840	0.000**
Luteinizing hormone LH (IU/L)	6.518±5.110	3.622±3.887	0.000**
Prolactin PRL (mIU/L)	420.110±643.568	318.754±209.857	0.156
Estradiol E2 (pmol/L)	220.461±170.960	136.386±90.434	0.000**
Progesterone	1.182±2.133	0.664±0.624	0.029*

**Note:** * P < 0.05, highly statistically significant difference; ** *P* < 0.01statistically significant difference.

**Table 3:** Clinical characteristic weight coefficients.

**Clinical Features**	**Weight Coefficient**
Ovulation-stimulating hormone FSH (IU/L)	-0.163288094
Luteinizing hormone LH (IU/L)	-0.085113266
BMI index number	-0.176691693
Age at bone age	-0.166459187
Estriol E2 (pmol/L)	-0.273441868

**Table 4:** Analysis of predictive performance of clinical models.

-	AUC (95%CI)	Accuracy	Sensitivity	Specificity
Training set	0.789 (0.7247-0.8524)	0.7778	0.2373	0.9753
Test set	0.844 (0.7487-0.9391)	0.7684	0.1583	1
External validation set	0.846 (0.732-0.9603)	0.766	0.7619	0.7692

**Table 5:** Differences in pituitary MRI morphology between IPPC and PT groups.

-	**ICPP (N=231)**	**PT (N=85)**	P price
**Shape of the Pendulum**	-	-	0.000**
Hollow	16 (6.9%)	12 (14.1%)	
Even	29 (12.6%)	31 (36.5%)	
Bulge	186 (80.5%)	42 (49.4%)	

**Note:** *P ** *P* < 0.01statistically significant difference.

**Table 6:** The omics characteristics and weight coefficients of T_1_WI vector + anterior pituitary in coronal view and T_1_WI vector in posterior pituitary in sagittal view.

**Imaging Omics Features**	**Weight Coefficient**
sqrroot_firstorder_90Percentile_T_1_WI Sagittal Posterior Pituitary	0.225
logarithm_glszm_ZonePercentage_T_1_WI Sagittal anterior pituitary	0.0723
wavelet-HLL_firstorder_RootMeanSquared_T_1_WI Sagittal pituitary anterior lobe	0.0354
Wavelet-LLH_ngtdm_Contrast_T_1_WI Sagittal view of anterior pituitary	0.0305
Wavelet-based HHL_gldm dependence non-uniformity normalized T_1_WI sagittal view of the anterior pituitary	0.0279
Wavelet-LLH_glrlm_RunEntropy_T_1_WI Sagittal anterior pituitary	-0.0209
lbp-3D-k_glcm_JointEntropy_T_1_WI Sagittal pituitary posterior lobe	-0.0457
Wavelet-LHH_glcm_ClusterShade_T_1_WI Coronal Position Hypophysis Anterior Lobe	-0.1108
gradient_firstorder_Skewness_T_1_WI Coronal pituitary anterior lobe	-0.1344
Wavelet-HLH_glcm_Correlation_T_1_WI Correlation T_1_WI Correlation T_1_WI Correlation T_1_WI Correlation T_1_WI Correlation T_1_WI	-0.359
originalShape_minorAxisLength_T_1_WICoronal pituitary anterior lobe	-0.3847
original.Shape_MajorAxisLength_T_1_WI Sagittal anterior pituitary	-0.5916
lbp-3D-m1_glrlm_RunEntropy_T_1_WI Sagittal anterior pituitary	-1

**Table 7:** Analysis of predictive performance of radiomics model.

-	AUC (95%CI)	Accuracy	Sensitivity	Specificity
**Training set**	-	-	-	-
RAD-AL1	0.814 (0.7436-0.8837)	0.8009	0.3898	0.9506
RAD-AL2	0.785 (0.7132-0.8562)	0.8009	0.339	0.9691
RAD-AL	0.807 (0.7337-0.8796)	0.8145	0.4237	0.9568
RAD-PL	0.725 (0.6538-0.7969)	0.7511	0.0678	1
RAD-Total	0.846 (0.7848-0.9078)	0.81	0.3729	0.9691
**Test set**	-	-	-	-
RAD-AL1	0.844 (0.7527-0.9352)	0.7895	0.3462	0.9565
RAD-AL2	0.799 (0.7032-0.8943)	0.7474	0.1154	0.9855
RAD-AL	0.867 (0.7924-0.9422)	0.8	0.3846	0.9565
RAD-PL	0.598 (0.4708-0.7243)	0.7368	0.0385	1
RAD-Total	0.854 (0.7786-0.9293)	0.7684	0.2692	0.9565
**External validation set**	-	-	-	-
RAD-AL1	0.848 (0.7394-0.9561)	0.7234	1.0	0.5357
RAD-AL2	0.803 (0.6704-0.9348)	0.766	0.7895	0.75
RAD-AL	0.8571 (0.7535-0.9607)	0.7021	1.0	0.5
RAD-PL	0.5564 (0.3879-0.7249)	0.5957	0.1053	0.9286
RAD-Total	0.827 (0.7079-0.9463)	0.7234	1.0	0.5357

**Table 8:** Differences between RAD-Total model and single sequence and fusion imaging omics models.

-	Training Set		Validation Set	
	z Grade	*P Price*	z Grade	*P Price*
RAD-AL1 model	2.191	0.028*	0.338	0.736
RAD-AL2 model	2.64	0.008*	1.305	0.192
RAD-AL model	2.431	0.015*	-0.514	0.607
RAD-PL model	2.749	0.006**	3.496	0.000**

**Note:** * P < 0.05, highly statistically significant difference; ** *P* < 0.01statistically significant difference.

**Table 9:** Analysis of predictive performance of clinical omics fusion model.

-	AUC (95%CI)	Accuracy	Sensitivity	Specificity
**Training set**	-	-	-	-
Clinical+RAD-AL1	0.8485 (0.7875-0.9095)	0.8281	0.5254	0.9383
Clinical+RAD-AL2	0.825 (0.7599-0.89)	0.8054	0.3898	0.9568
Clinical+RAD-AL	0.8481 (0.7666-0.9095)	0.8416	0.5254	0.9568
Clinical+RAD-PL	0.8306 (0.7757-0.8855)	0.7919	0.322	0.963
Clinical+RAD-Total	0.871 (0.8167-0.9242)	0.8281	0.4915	0.9506
**Test set**	-	-	-	-
Clinical+RAD-AL1	0.9025 (0.8419-0.963)	0.8316	0.5385	0.942
Clinical+RAD-AL2	0.9097 (0.8484-0.971)	0.8211	0.4231	0.971
Clinical+RAD-AL	0.913 (0.8549-0.9712)	0.8211	0.5	0.942
Clinical+RAD-PL	0.9091 (0.8439-0.9744)	0.8211	0.3462	1
Clinical+RAD-Total	0.944 (0.902-0.9854)	0.8632	0.5769	0.8632
**External validation set**	-	-	-	-
Clinical+RAD-AL1	0.8828 (0.7858-0.9797)	0.766	0.9524	0.6154
Clinical+RAD-AL2	0.8333 (0.7075-0.9592)	0.8085	0.9048	0.7308
Clinical+RAD-AL	0.8864 (0.7897-0.9832)	0.7447	1	0.5385
Clinical+RAD-PL	0.7436 (0.5956-0.8916)	0.766	0.9048	0.6538
Clinical+RAD-Total	0.863 (0.7526-0.9727)	0.7447	1.0	0.5385

**Table 10:** Characteristics and weight coefficients of the clinical-RAD Total model.

Feature Name	Weight Coefficient
Score-RAD total	1
BMI index number	-0.0293
Bone Age Index (BAI)	-0.0375
Follicle-stimulating hormone (FSH)	-0.1942

## Data Availability

The datasets used during the current study are available from the corresponding authors [F.Y] and [S.L.] on reasonable request.

## LIST OF ABBREVIATIONS

ACC: Accuracy
AUC: Area under curve
BAI: Bone age index
BMI: Body Mass Index
CPP: Central precocious puberty
DCA: Decision Curve Analysis
E2: Estradiol
FSH: Follicle-stimulatinghormone
GnRH: Gonadotropin releasing hormone
GnRHa: Gonadotropin-releasing hormone agonist
HPGA: Hypothalamic pituitary gonadal axis
ICC: Intraclass correlation coefficient
ICPP: Idiopathic centralprecocious puberty
LASSO: Least absolute shrinkage and selection operator
LH: Luteinizing hormone
MRI: Magnetic resonance imaging
NB: Net Benefit
PP: Precocious puberty
PPP: Peripheral precocious puberty
PT: Premature thelarche
ROC: Receiver Operating Characteristic
ROI: Region of Interes
SEN: Sensitivity
SPE: Specificity
T1WI: T1 weighted image,T1WI
TE: Echo time

## STANDARDS OF REPORTING

STROBE guidelines were followed.
